# Prediction of Depression Scores From Aerobic Fitness, Body Fatness, Physical Activity, and Vagal Indices in Non-exercising, Female Workers

**DOI:** 10.3389/fpsyt.2019.00192

**Published:** 2019-04-12

**Authors:** Laís Tonello, Iransé Oliveira-Silva, André Ricarte Medeiros, Arthur Ney Alves Donato, Felipe Barreto Schuch, Lars Donath, Daniel Boullosa

**Affiliations:** ^1^Educação Física, Universidade de Gurupi, Gurupi, Brazil; ^2^Escola de Saúde e Medicina, Universidade Católica de Brasília, Brasília, Brazil; ^3^Educação Física, UniEVANGÉLICA - Centro Universitário de Anápolis, Anápolis, Brazil; ^4^Mestrado em Saúde e Desenvolvimento Humano, Universidade La Salle Canoas, Canoas, Brazil; ^5^Department of Intervention Research in Exercise Training, German Sport University, Cologne, Germany; ^6^Sport and Excercise Science, James Cook University, Townsville, QLD, Australia

**Keywords:** depression, physical activity, autonomic control of HR, body composition, physical fitness, women

## Abstract

**Background:** Depression is associated with a decreased cardiorespiratory fitness, and physical activity [PA] levels, higher rates of obesity, and dysfunction in autonomic control of heart rate [HR]. However, these parameters were mostly recorded with indirect methods. Thus, the aim of the current study was to investigate the relationships between depression scores and objective measures of body fatness, autonomic indices (i.e. HRV and HRR), cardiorespiratory fitness and PA levels; and subsequently to present the best predictive models of depression scores for this population, based on these variables.

**Methods:** Thirty-five non-exercising women (26–43 years; maximal oxygen consumption [VO_2_max] ~ 17.4–38.3 mL/kg/min) volunteered for participation in this study. All participants responded to the Beck Depression Inventory [DBI] and were evaluated for body mass index [BMI], percentage of body fat, sum of skinfolds, and VO_2_max. Subsequently, over four consecutive days, an orthostatic test and a submaximal exercise on a cycle ergometer were performed to record HRV and HRR, respectively. In addition, incidental PA was recorded during 5 consecutive days using accelerometers.

**Results:** depression scores were related to VO_2_max (*r* = −0.446, *p* = 0.007) and the sum of skinfolds (*r* = 0.434, *p* = 0.009). Several stepwise multiple linear regression models were performed and only VO_2_max was revealed as an independent predictor of the Beck scores (ß = −0.446, *R*^2^ = 0.199, *p* = 0.007).

**Conclusion:** The present study revealed that VO_2_max and the sum of skinfolds were moderately related to depression scores, while VO_2_max was the only independent predictor of depression scores in female workers.

## Introduction

Depression is a multifactorial disease that affects 322 million people worldwide (1). Between 2005 and 2015, there was an increase of more than 18% in the number of cases (1). The global prevalence is 4.4%, however, women suffer more from the disease, with 5.1% compared to 3.1% of men (1). In Brazil, depression affects 7.6% of the population, which represents about 11.2 million people, with a prevalence of 10.9% in women and 3.9% in men (2), therefore ranking Brazil as the 5th country in the world in depression prevalence (2).

Depression is a prevalent risk factor for the development of cardiovascular and metabolic (3–5) diseases (CVDs) (6–10). For example, people with depression presents an increased risk (relative risk [RR] = 1.72, 95% CI 1.48–2.00) of developing any cardiovascular disease when compared to non-depressed controls. In addition, people with subsyndromal depressive symptoms are already in increased risk for developing cardiovascular disease (11). Although the underlying mechanisms of this relationship are not fully understood, there are several factors that act bidirectionally and independently, increasing both depression and CVDs risk, including aerobic capacity (12), obesity (13), physical activity (14), and heart rate variability (HRV) (15).

Cardiovascular diseases are the main cause of death in women (7, 16) and present an inverse relationship with cardiorespiratory fitness (17, 18). Moreover, cardiorespiratory fitness is inversely related with depressive symptoms severity, regardless depression diagnosis, and this association has been demonstrated to be moderated by sex (12). Thus, people with depression have decreased fitness when compared to people without depression (19, 20). Moreover, a possible relationship between depression, lack of exercise and cerebrovascular disease comorbidity has also been suggested (21).

Overweight and obesity has been associated with depression, in addition to be an aggravating factor for overall health status (13, 22–24). The association between obesity and depression is bidirectional: depressed people are at an increased risk of being obese and, in turn, obese people have a risk of developing depression (25). Interestingly, although the percentage of fat increases the risk for developing depressive symptoms, this association disappears when adjusted for cardiorespiratory fitness levels in both men and women (23), with some sex differences identified in the association between obesity and depression (22).

Individuals with depression have a decreased HRV when compared to healthy controls (15, 26–29), and this inverse association between HRV and depressive symptoms has also shown different sex related interactions (30). The severity of depressive symptoms is an independent factor for decreased HRV and vagal tone in patients with unipolar and bipolar depression (31). The reduction in HRV is commonly associated with cardiovascular morbidity and mortality, thus, depressive symptoms, decreased HRV, and risk of developing cardiovascular disease are closely related (32). In addition, autonomic activity could be also assessed with HRR, which is an index of vagal reactivation after different exercises (33–38). Heart rate recovery has been also found to be inversely related with depression scores (39, 40).

Finally, PA has been previously associated with all of the aforementioned factors and has been recommended as a complement to pharmacotherapy for the treatment of depression (41). Previous studies have also shown that the association of PA with other forms of treatment is a determinant for a decrease in depression scores (42). People with higher PA levels have 17% less risk of developing depression (43). However, these previous studies did not account for the difference between PA (i.e., any movement of the body that is produced by muscle contraction and which increases energy expenditure above basal levels) and physical exercise (i.e., a repetitive structured PA designed to improve or maintain one or more components of the physical condition) (44–48).

Although several studies have independently investigated the relationship between the above-mentioned variables (i.e., PA, HRV, HRR, body fatness) and depression scores, to the best of our knowledge, there are no studies investigating the relationship of all these physical and physiological variables, and their interactions, with depression scores in a homogenous sample of women. Thus, the aim of the current study was to investigate the relationships between depression scores and objective measures of body fatness, autonomic indices (i.e., HRV and HRR), cardiorespiratory fitness and PA levels; and subsequently, to present the best predictive models of depression scores for this population, based on these variables.

## Methods

### Participants

All participants were employees of two private Universities in Brazil. All of them worked in the cleaning service or administrative staff. The inclusion criteria were: absence of health related problems or physical conditions; no medications that could interfere with independent and dependent variables; and no practice any form of regular exercise at the time of the study. Thus, 35 adult female workers, aged 34.5 ± 5.1 years (26–43 years), were finally included in this study. All of them were classified as irregularly active or sedentary by the short version of the international PA questionnaire (IPAQ) (49), a short validated questionnaire with eight questions regarding the weekly time devoted to different levels of PA and sedentary time. This research was approved by the ethics committee of the Catholic University of Brasília and all volunteers signed the consent form after detailed explanation of procedures.

### Study Design

**Figure 1:**
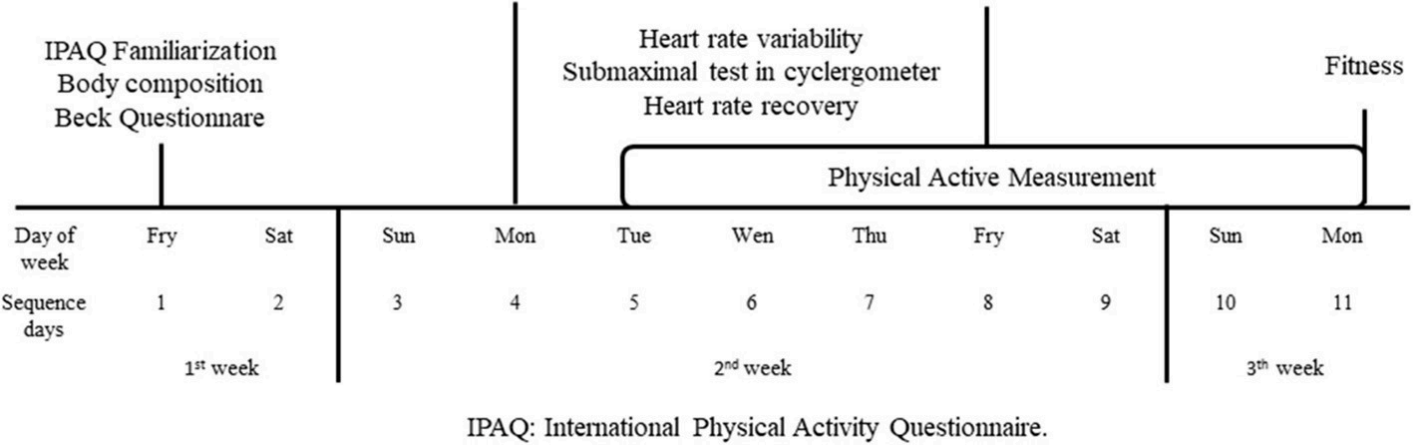
Schedule of evaluations.

All participants were evaluated for 14 days following the methods of a previous study (36). The schedule of evaluations is presented in Figure 1. During the first week, participants were familiarized with all the procedures, completed the IPAQ and Beck Depression inventory (BDI) (50), and were evaluated for body fatness and cardiorespiratory fitness. During the second week, participants went to the laboratory on a daily basis and were evaluated for HRV and HRR. PA was recorded over 7 days, but only measures from five days (Wednesday to Sunday) were used for further analyses (36). During the third week, participants completed the cardiorespiratory fitness test.

### Measures

#### Body Composition

Body mass (kg) was evaluated using a digital scale (Model 05, G-Tech®, China), and height (cm) through a stadiometer (Sanny®, ES2040, Brazil) to determine body mass index (BMI). Adiposity was evaluated using the sum of the skinfolds (∑S) (i.e., pectoral, mid axillary, abdominal, supra iliac, thigh, triceps and subscapular) (47), with % of body fatness determined with the equations of Jackson and Pollock (51) and (52).

#### Cardiorespiratory Fitness

Maximum oxygen consumption (VO_2_max) was assessed as a measure of cardiorespiratory fitness, during a maximal incremental test on a cycle ergometer (Lode Excalibur, Lode, Netherlands; or Monark model 8348, Monark, Sweden). The test started with a load of 0 W and thereafter it was increased at a rate of 20 W·min^−1^, maintaining a constant cadence of 60 rpm. HR was continuously recorded using a telemetric monitor (POLAR Electro Oy, Finland), while ventilatory parameters were assessed breath-by-breath with a metabolic cart (Metalyzer 3B, Cortex, Germany) that was previously calibrated following manufacturer's instructions.

#### Beck Depression Inventory

The BDI is a valid questionnaire with 21 questions that include cognitive and affective behavioral symptoms and attitudes related to depression. The subjects selected the sentence that best described their answer during their previous two weeks. The sum of the final score can be from 0 to 63 (53). All the questionnaires were individually applied by the same clinician, in a closed room, under a calm environment and without the possibility of any interruption by other people. The cut-off scores for the BDI are: none or minimal depression with <10; mild to moderate depression with 10–18; moderate to severe depression with 19–29; and severe depression with 30–63.

#### Autonomic Indices

The evaluation of HRV was performed during four consecutive days, through an orthostatic test, performed in the laboratory (07:00–08:00 a.m.) under standard environmental conditions. During the orthostatic test, participants remained seated for 3 min followed by 4 min in standing position. All HR recordings were obtained using a HR monitor (RS800cx, Polar Electro Oy, Finland) previously validated for this purpose (54). Subsequently, HR data were transferred to a computer and filtered using the customer's specific software (Polar ProTrainer® 5, Polar Electro Oy, Finland). If a record presented error >5%, it was discarded (55). After visual inspection, filtering and correction, the data was transferred and analyzed on a specific HRV analysis software (Kubios 2.2, The Biomedical Signals Analysis Group, Finland). Vagal modulation was assessed via the root mean square of successive R-R intervals differences (RMSSD) during the last 2 min of each position (RMSSD_seated_ and RMSSD_standing_, respectively). Following a previous study (36), we used the average values over the four days of evaluation.

For HRR determination, the volunteers performed a 6-min submaximal cycle ergometer exercise (model 8,348, Monark, Sweden) immediately after HRV evaluations. At the end of the exercise, the volunteers remained seated and relaxed on the cycle ergometer for 5 min for determination of HRR at 1 min (HRR_1min_) and at 5 min (HRR_5min_). As in the case of HRV analyses, we used the average values over the four days of evaluation (36).

#### Incidental Physical Activity

The PA was recorded with an accelerometer (GT1M, Actigraph, USA), being considered the values of five consecutive days, which is enough to reflect weekly AF patterns in adults (56). The devices were placed on the right hip of participants and PA was recorded continuously, except during bathing, sleeping and the cycle ergometer submaximal exercise. Following the procedures of a previous study (36), the parameters analyzed were: steps per day (Steps), and moderate to vigorous PA (MVPA).

### Statistical Analyses

The distribution of the data was verified for normality with Shapiro Wilk's test. Scaterplot of the predicted values vs. the standard residuals of the variables were verified for homoscedasticity and presence of outliers. To achieve the statistical assumptions above mentioned, some variables were log-transformed. Subsequently, Pearson product correlation coefficient was used to verify independent associations with the dependent variables, and for multicolinearity of the independent variables. Multicolinearity was considered when independent variables presented correlations with *r* ≥ 0.7. Then, a stepwise regression was applied with Beck scores as the dependent variable, and with all other parameters as independent variables. Thereafter, due to the number of subjects in the final sample, some multiple linear regression models were constructed using a combination of up to four variables, one from each different domain (e.g. body composition, aerobic fitness, PA, and autonomic indices) to achieve the better model of prediction of depression scores, preserving an sufficient statistical power for the analyses. A 5% level of significance was adopted.

## Results

Characteristics of adiposity, aerobic fitness, PA levels, autonomic indices, and depression scores of participants are presented in Table 1.

**Table 1:** Demographic characteristics and physical activity levels of participants.

**Parameters**	**Mean ± SD**	**Range**
BDI-scores	12.3 ± 7.1	0.0–28.0
Age (years)	34.5 ± 5.1	26–43
Body mass (kg)	65.5 ± 13.7	48.6–110.0
Height (m)	1.57 ± 0.09	1.28–1.68
BMI (kg·m^−2^)	26.01 ± 4.9	18.1–36.5
Body fat (%)	33.98 ± 6.7	19.9–43.1
ΣS (mm)	206.2 ± 55.8	102–293
VO_2_max (mL·kg^−1^·min^−1^)	25.2 ± 5.2	17.4–38.3
Steps·day^−1^ (Steps)	8597 ± 3204	3257–15464
MVPA (min·day^−1^)	41.5 ± 22.4	7–84.1
RMSSD_seated_ (ms)	25.8 ± 9.0	8.6–46.5
RMSSD_upright_ (ms)	17.9 ± 5.7	9.8–31.9
HRR_1min_ (bpm)	31.3 ± 7.2	18.7–49.7
HRR_5min_ (bpm)	53.6 ± 5.2	45.5–64.7

*SD, standard deviation; BDI-scores, beck depression inventory scores; BMI, body mass index; Body Fat%, percentage of body fatness; ΣS, sum of 7 Skinfold; VO_2max_, maximum oxygen consumption; Steps·day^−1^, mean daily steps; MVPA, mean daily moderate to vigorous PA of 4 days of data collection; RMSSD, root Mean Square of the differences between successive R peak intervals; HRR_1min_, heart rate recovery of the first minute; HRR_5min_, heart rate recovery of the fifth minute*.

**Figure 2:**
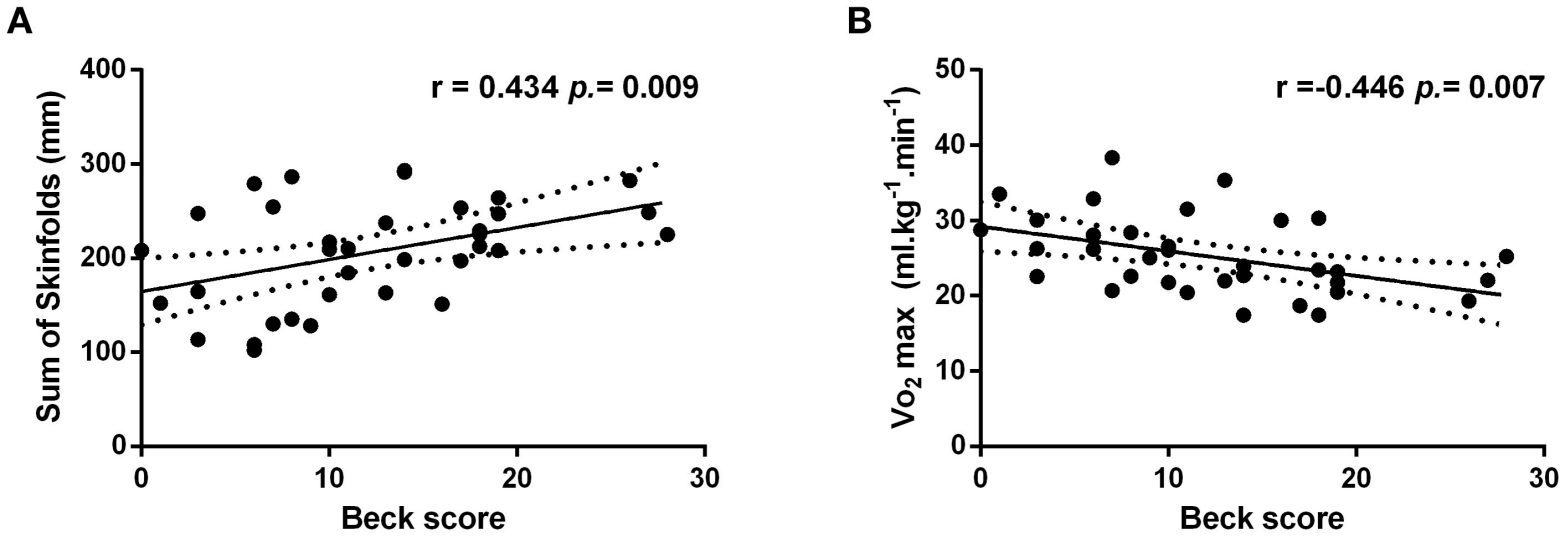
Correlation between Beck scores and the sum of skinfolds **(A)** and VO_2_max **(B)**.

Significant correlations were found between some independent variables (see Table 2). Although multicollinearity was observed in some of the independent variables, none of the regression models included those variables simultaneously. Besides that, ΣS and VO_2_max were significantly and independently correlated with the dependent variable (Beck scores), as reported in Figures 2A,B, respectively.

**Table 2:** Correlation matrix of dependent and independent variables (*n* = 35).

	**1**.	**2**.	**3**.	**4**.	**5**.	**6**.	**7**.	**8**.	**9**.	**10**.
1. Beck scores	−	−0.446^‡^	0.351	0.435^‡^	0.056	0.173	0.136	0.237	0.188	0.001
2. VO_2_max	−0.446^‡^	−	−0.528^‡^	−0.642^‡^	−0.038	0.041	−0.088	−0.215	−0.235	−0.138
3. Body Fat (%)	0.351	−0.528^‡^	−	0.816^‡^	0.243	0.124	0.042	0.035	0.183	0.211
4. ∑S	0.435^‡^	−0.642^‡^	0.816^‡^	−	0.110	0.060	0.156	0.163	0.198	0.090
5. Steps·day^−1^	0.056	−0.038	0.243	0.110	−	0.731^‡^	0.201	0.090	0.567^‡^	0.536^‡^
6. MVPA	0.173	0.041	0.124	0.060	0.731^‡^	−	0.319	0.143	0.383^*^	0.123
7. RMSSD_seated_	0.136	−0.088	0.042	0.156	0.201	0.319	−	0.564^‡^	0.419^*^	0.171
8. RMSSD_standing_	0.237	−0.215	0.035	0.163	0.090	0.143	0.564^‡^	−	0.435^‡^	0.199
9. HRR_1min_	0.188	−0.235	0.183	0.198	0.567^‡^	0.383^*^	0.419^*^	0.435^‡^	−	0.707^‡^
10. HRR_5min_	0.001	−0.138	0.211	0.090	0.536^‡^	0.123	0.171	0.199	0.707^‡^	−

Beck, Beck inventory depression scores; VO_2max_, maximum oxygen uptake; Fat%, Body percentage of fatness; ∑S, sum of skinfolds; Steps/Day, mean daily steps; MVPA, minutes per day on moderate to vigorous PA; sRMSSD, Root Mean Square of the differences between successive R peak intervals recorded in seated condition; uRMSSD, Root Mean Square of the differences between successive R peak intervals recorded in the standing condition; 1minHRR, one minute heart rate recovery; 5minHRR, five minutes heart rate recovery.

* p < 0.05;

‡ p < 0.01.

The stepwise multiple linear regression model selected only VO_2_max as a predictor of Beck scores, with standard ß = −0.446, *R*^2^ = 0.199, and *p* = 0.007. Then, another 16 models were tested, and the better model achieved *R*^2^ = 0.278 and *p* = 0.039, including VO_2_max (std.ß = −0.283, *p* = 0.180), SS (std.ß = 0.224, *p* = 0.279), MVPA (std.ß = 0.154, *p* = 0.338), and RMSSD_standing_ (std.ß = 0.117, *p* = 0.472). Although that model reached statistical significance, none of the independent variables adopted were found to be significant independent predictors of Beck scores. As expected, for other models tested, the only variable who independently predicted Beck scores was VO_2_max. The models are presented in Table 3.

**Table 3:** Regression analysis for variables predicting Beck Scores (*n* = 35).

	**Regression**	**VO_**2max**_**	**Body Fat (%)**	**∑S**	**Steps·day^**−1**^**	**MVPA**	**RMSSDseated**	**RMSSDstanding**	**HRR_**1min**_**	**HRR_**5min**_**
**Model**	**R^**2**^ (*p*.)**	**Std*.B* (*p*.)**	**Std*.B* (*p*.)**	**Std*.B* (*p*.)**	**Std*.B* (*p*.)**	**Std*.B* (*p*.)**	**Std*.B* (*p*.)**	**Std*.B* (*p*.)**	**Std*.B* (*p*.)**	**Std*.B* (*p*.)**
1-Stepwise	0.199 (0.007)^**^	−0.446 (0.007)^**^	-	-	-	-	-	-	-	-
02-Insert	0.227 (0.092)	−0.350 (0.077)	0.166 (0.404)	-	−0.018 (0.915)	-	0.102 (0.539)	-	-	-
03-Insert	0.242 (0.073)	−0.330 (0.095)	0.173 (0.380)	-	–0.010 (0.951)	-	-	0.158 (0.335)	-	-
04-Insert	0.226 (0.095)	−0.332 (0.104)	0.170 (0.396)	-	−0.063 (0.760)	-	-	-	0.115 (0.576)	-
05-Insert	0.227 (0.092)	−0.374 (0.060)	0.162 (0.414)	-	0.067 (0.734)	-	-	-	-	−0.121 (0.535)
06-Insert	0.248 (0.065)	−0.380 (0.054)	0.128 (0.504)	-	-	0.157 (0.364)	0.047 (0.780)	-	-	-
07-Insert	0.263 (0.051)	−0.358 (0.068)	0.138 (0.468)	-	-	0.150 (0.362)	-	0.131 (0.423)	-	-
08-Insert	0.246 (0.067)	−0.385 (0.056)	0.125 (0.515)	-	-	0.169 (0.344)	-	-	0.010 (0.956)	-
09-Insert	0.257 (0.057)	−0.393 (0.044)	0.143 (0.458)	-	-	0.184 (0.262)	-	-	-	−0.106 (0.518)
10-Insert	0.242 (0.073)	−0.286 (0.179)	-	0.239 (0.264)	0.004 (0.981)	-	0.073 (0.659)	-	-	-
11-Insert	0.255 (0.058)	−0.258 (0.224)	-	0.245 (0.245)	0.006 (0.968)	-	-	0.140 (0.393)	-	-
12-Insert	0.243 (0.072)	−0.264 (0.222)	-	0.250 (0.241)	−0.037 (0.852)	-	-	-	0.097 (0.630)	-
13-Insert	0.244 (0.071)	−0.302 (0.160)	-	0.242 (0.256)	0.072 (0.708)	-	-	-	-	−0.101 (0.600)
14-Insert	0.266 (0.049)^*^	−0.307 (0.145)	-	0.224 (0.286)	-	0.165 (0.329)	0.022 (0.896)	-	-	-
15-Insert	0.278 (0.039)^*^	−0.283 (0.180)	-	0.224 (0.279)	-	0.154 (0.338)	-	0.117 (0.472)	-	-
16-Insert	0.265 (0.049)^*^	−0.306 (0.154)	-	0.169 (0.334)	-	0.169 (0.334)	-	-	0.006 (0.972)	-
17-Insert	0.272 (0.043)^*^	−0.321 (0.130)	-	0.225 (0.279)	-	0.183 (0.258)	-	-	-	−0.086 (0.592)

VO_2max_, maximum oxygen consumption; Body Fat (%), body percentage of fatness; ∑S, sum of 7 Skinfolds; Steps·day^−1^, mean daily steps; MVPA, mean daily moderate to vigorous PA daily mean for the 4 days data collection; RMSSDstanding, HR recorded in upright position; RMSSDseated, HR recorded on seated position; HRR_1min_, heart rate recovery of the first minute after de exercise; HRR_5min_, heart rate recovery of five minutes after de exercise.

* p < 0.05;

** *p < 0.01*.

## Discussion

To the best of our knowledge, this is the first study evaluating the relationship and interaction of depression scores with the autonomic control of HR, PA, cardiorespiratory fitness and body fatness in the same sample. Several models were tested to investigate what variables were able to predict depression scores (see Table 3). Thus, we observed that in women who do not exercise regularly, the depressive symptoms are related to the level of cardiorespiratory fitness and body fatness. However, the only variable able to predict independently depression scores was VO_2_max. In contrast, and contrary to our hypotheses, incidental PA or autonomic control of HR were not associated with depression scores.

As shown in previous studies (12, 19, 23, 57–60), depression scores demonstrated an inverse association with cardiorespiratory fitness. In addition, our main finding is that cardiorespiratory fitness is the best predictor of depressive symptoms independently of body fatness, confirming the previous findings of the prospective study of Becofsky et al. (23). According to our model, changes in VO_2_max values may explain up to 19.9% of the variance in the depression scores, therefore, an increase of ~2 mL·kg^−1^·min^−1^ of VO_2_max would imply ~1.2 points less in the depression score. While the relationship between aerobic fitness and depression has been previously suggested to be mediated by PA, this is not the case in the current study. In this regard, exercise has been previously demonstrated to influence depression independently of its intensity (61), perhaps implying that the impact of aerobic fitness *per se* on depression scores and symptoms could be mediated by other factors. Thus, we may suggest the possible association between aerobic fitness and other biological mediating factors related to mental health and cognition. For instance, previous reports have found a relationship between VO_2_max, inflammation, and brain-derived neurotrophic factor (BDNF) in different populations (62–64), factors also recognized to be related to depression (65). Therefore, future studies are needed to better clarify the mechanistic link between VO_2_max, other aerobic fitness parameters and depression.

In our study, body fat did not correlate with depression scores, however, they were associated with the sum of skinfolds. The association between body composition parameters with depression scores has presented contradictory results in literature, a fact that can be attributed to differences in methods to characterize overweight and obesity, in addition to the existence of a number of confounding variables. For instance, previous studies identified an association between BMI and depression scores (13, 24, 66). In addition, women with % of body fatness greater than 30 were more likely to develop depressive symptoms (23). Our results may be also explained by the fact that % of body fatness is an estimated measure, while the sum of skinfolds is an objective measure. Future studies should consider these aspects when verifying these associations in further studies.

Our data did not reveal any relationship between objective measured PA and depression, which is in contrast to previous literature (14, 43, 67–72)]. The lack of association may be attributed to the different protocols used for evaluation of depression scores and PA levels (e.g., selection of cut-off points for different PA levels). However, a strength of the current study is the use of objective measures of PA along with the isolation of incidental PA from exercise, as participants who exercised regularly were not included in the current investigation. Therefore, it may be suggested that this finding is not necessarily in contradiction with previous studies that mostly used indirect measures of PA levels, while they did not differentiate between exercise and overall incidental PA levels. In this respect, it should be pointed out that women in the current study averaged 41 min/day of moderate to vigorous PA. Meanwhile, previous studies showed that 30 min/day, three times a week, of moderate PA is enough to reduce the risk of developing depression by 22% (43). In addition, although the number of daily steps has not been shown to be associated with depressive symptoms, mean values in the current study are above the recommendations of 6,500-8,500 steps for special populations (73, 74). In this regard, given that our sample was composed of female workers, and that probably most PA was performed at work, future studies differentiating between leisure time and occupational PA are needed as these two PA dimensions have both been demonstrated to influence cardiovascular health in different ways (75).

The use of PA and physical exercise for the treatment of depression is currently increasing (42, 73, 74, 76–79). The improvement of cardiorespiratory fitness in a short period of time with regular physical exercise could be suggested as an effective means for this purpose (59). However, PA performed in daily life could also be important in order to improve levels of cardiorespiratory fitness (80). Stimulating the participation of individuals with depression in physical exercise programs can be an effective treatment alternative, given the low barriers associated and their effectiveness for improving depressive symptoms (81). Recently, Busch et al. (82) evaluated the preferences and barriers of exercise for the treatment of depression. The results of this study (82) indicated that the lack of motivation and fatigue are the main barriers to exercise (82). Therefore, in order to identify the best exercise model and the amount of incidental PA for the treatment of depression, it is necessary to take into account the effectiveness of every exercise program to improve cardiorespiratory fitness, highlighting the inverse relationship between aerobic fitness and scores of depression.

Although, Busch et al. (82) addressed some characteristics of an exercise model for this population, it is clear that there is no unanimity for the choice of intensities, duration and type of exercise. For instance, high-intensity training could be an interesting option as it has been shown to be effective for improving VO_2_max and other health related parameters in a short period of time in different populations (83–85). Therefore, PA and physical exercise should be evaluated separately or in conjunction in future investigations to determine the better dose-response for the improvement of cardiorespiratory fitness in people with depression.

Previous studies have suggested that autonomic control of HR is directly related to depression scores (15, 29, 86–89). In contrast, the current study did not reveal any association between HRV or HRR and depression scores. Cardiac autonomic modulation, assessed with HRV, besides important associations with cardiovascular diseases (90), has also been suggested as an indicator of the body's ability to adapt to stress (91), thus playing an important role in the interpretation and response to emotional stimuli (92). In the case of people with depression, the reduction of HRV has been demonstrated to be linked to the severity of the disease (15, 29, 89), with sympathetic predominance and reduction of parasympathetic activity, resulting in reduced HRV (86, 87). In this regard, PA and exercise have been highlighted for the improvement of HRV (93, 94). This adaptation is thought to be related to improvements in VO_2_max (80). However, the current study did not reveal any relationship between PA, HRV and VO_2_max. In contrast, the current results showed some relationships between HRV, HRR, and PA, which is partially in agreement with a previous study of our group (36). Therefore, given the unique characteristic of the current study with objective measures of these parameters, and with participants not involved in physical exercise, it may be speculated that the inclusion of participants practicing exercise would result in higher VO_2_max levels, and thus some correlations between aerobic fitness and HRV.

This study is not free from limitations. First, given the cross-sectional design, causal relationships cannot be established. Second, the low number of participants may be another limitation. However, the use of objective measures for all the parameters investigated, with average values over various days for HRV and HRR, in conjunction with stringent criteria for inclusion of adult female participants, reduce the noise importantly when compared to previous studies looking for similar relationships with greater but very heterogeneous samples with unknown confounders. Finally, from a biopsychosocial perspective, other factors such as socioeconomic status were not recorded, therefore, further studies should verify the possible interaction between physical and physiological variables with other psycho-social influences on depression scores and symptoms (95).

## Conclusions

The present study revealed that VO_2_max and the sum of skinfolds were moderately related to depression scores, while VO_2_max was the only independent predictor of depression in female workers. However, incidental PA, HRV, and HRV were not related to depression scores in this small but homogenous sample. Future studies with greater samples should identify the best models of physical exercise, while monitoring levels of incidental PA, for promoting improvements in cardiorespiratory fitness and other health-related parameters as body composition and autonomic indices. The mechanistic link between aerobic fitness and depression remains to be clarified.

## Ethics Statement

Ethical approval was obtained from the Ethics Committee of the Catholic University of Brasília. In addition, a consent from was obtained from every participant.

## Author Contributions

LT and DB: study design. LT, IO-S, and ARM: data collection. LT, DB, IO-S, and ARM: data analyses. LT, IO-S, ARM, ANAD, FS, LD, and DB: interpretation of the results, manuscript writing, and approved the final manuscript version.

### Conflict of Interest Statement

The authors declare that the research was conducted in the absence of any commercial or financial relationships that could be construed as a potential conflict of interest.
